# The Performance of Three-Sample Qualitative Immunochemical Fecal Test to Detect Colorectal Adenoma and Cancer in Gastrointestinal Outpatients: An Observational Study

**DOI:** 10.1371/journal.pone.0106648

**Published:** 2014-09-08

**Authors:** Dong Wu, Han-Qing Luo, Wei-Xun Zhou, Jia-Ming Qian, Jing-Nan Li

**Affiliations:** 1 Department of Gastroenterology, Peking Union Medical College Hospital, Chinese Academy of Medical Sciences, Peking Union Medical College, Beijing, China; 2 Department of Pathology, Peking Union Medical College Hospital, Chinese Academy of Medical Sciences, Peking Union Medical College, Beijing, China; University Hospital Llandough, United Kingdom

## Abstract

**Background:**

Repeated qualitative fecal immunochemical test (qlFIT) is a clinical strategy widely used to detect lower gastrointestinal lesions, but its diagnostic power has not been assessed in opportunistic screening for colorectal neoplasia.

**Objective:**

This study aimed to determine the performance of three-sample qlFIT in screening for colorectal cancer and its precursors in high-risk participants.

**Methods:**

513 gastrointestinal outpatients yielded three qlFITs before a standard colonoscopy. We evaluated the diagnostic value of one, two, and three positive qlFITs serving as the positivity threshold. The risk factors of colorectal neoplasia to yield positive qlFITs were also determined.

**Results:**

52 patients were diagnosed with colorectal cancer and 70 with advanced adenomatous polyp. For colorectal cancer, the sensitivity and specificity of one positive qlFIT were 90.4% and 53.8%, of two were 80.8% and 75.1%, and of three were 53.9% and 88.5%, respectively. For advanced adenomatous polyp, the sensitivity and specificity of one positive qlFIT were 81.4% and 54.2%, of two were 50.0% and 72.5%, and of three were 28.6% and 86.2%. Left-sided location (OR 2.50, 95%CI 1.26–4.95) and advanced histology of tumors (OR 3.08, 95%CI 1.58–6.01) were independently associated with positive qlFITs.

**Conclusions:**

Three-sample qlFIT is a reasonably good method to detect colorectal neoplasia in high-risk population. Tumors in the left side or with advanced pathological features are more likely to produce positive qlFITs.

## Introduction

Colorectal cancer (CRC) is the third most common cancer in men and the second in women worldwide, claiming 608,000 deaths in 2008 [1]. Although age-adjusted incidence of CRC has been in decline in the USA since 1985, it is worrisome to observe that CRC has increased rapidly in developing areas such as China over the last decades [2], [3]. The current CRC screening guidelines in the USA recommended several options including colonoscopy and computerized tomographic colonography (CTC) [4]–[6]. In China, however, limited medical resources have made it difficult to perform colonoscopy-based screening in average-risk population [7]. Instead, Chinese program is centered on fecal occult blood test, particularly in opportunistic screening among gastrointestinal outpatients.

In daily practice physicians often use periodic qualitative fecal immunochemical tests (qlFITs) with frequent intervals to detect lesions of the lower digestive tract. But the performance of this strategy has not been assessed in screening of colorectal neoplasia. In contrast, many investigators favored one-sample quantitative fecal immunochemical tests (qnFITs) as a screening or surveillance method, based on its transparency of a quantitative result, and the flexibility to adjust cutoff values toward different needs and resources [8]–[10]. But this approach has two drawbacks that merit improvement. First, as colorectal polyps tend to bleed intermittently, repeated measurements should be more sensitive than single test to detect them [11]–[14]. Second, the cost of qnFIT is much higher than that of qlFIT. Given that qlFIT is less expensive and more readily available than qnFIT, the screening power of qlFIT for colorectal neoplasia is of clinical and financial importance, especially for those with disadvantaged socioeconomic conditions. Some studies have confirmed the diagnostic effect of qlFITs for colorectal neoplasia, but it is less clear if such efficacy is comparable to that of qnFITs [15]–[18].

This study aimed to determine the utility of three-sample qlFIT of separate bowel movements in: (i) identifying the presence of significant neoplasms (CRC or advanced adenoma) in high-risk patients having a scheduled colonoscopy; (ii) determining the number of colonoscopies that would have been needed because of positive qlFITs; and conversely, the number of colonoscopies that could have been postponed and (iii) the potential risk factors of colorectal neoplasia to yield positive qlFIT results.

## Methods

**Ethics Statement**: The Ethics Committee of Peking Union Medical College Hospital approved the study protocol, and people who met the inclusion criteria provided written informed consent.

### Study design

This was a retrospective analysis of a prospective database. From June 2011 to December 2013, consecutive outpatients of the department of gastroenterology, Peking Union Medical College Hospital, who had been scheduled for a colonoscopy examination, were enrolled in this study. Indications for colonoscopy were determined according to the physician's judgment, including a history of colorectal polyp, family history of CRC, or symptoms related to the lower gastrointestinal tract. Participants who had a history of other diseases that may produce fecal blood, such as active diverticulitis, inflammatory bowel disease, Non-Hodgkin's lymphoma involving digestive tract, vasculitis, intestinal tuberculosis, angiodysplasia, and who had received surgical resection of any part of the large bowel, were excluded from the study. Exclusions also referred to those with hematuria or menstruation at that time and noncooperation with preparing three fecal tests. No dietary or medication restrictions were required before stool sample collection.

### Stool samples and qlFIT

Participants obtained three fecal samples at home on three separate days within one week prior to the colonoscopy. In three hours samples were sent to the central laboratory of our institute for a qlFIT assay (Acon Biotech (Hangzhou) Co., Ltd., Hangzhou, China). The test had a positive cutoff level of 50 ng/ml. The cost of each test was 15 RMB (about $2.5). The measurement was made with a sampling probe that was inserted into the stool until the groove of the probe was completely covered by the stool according to the manufacturer's instructions. The probe was then immediately inserted into the sampling tube and the result was read at 5 min.

### Colonoscopy and histology

Endoscopists with at least five years of experience performed the colonoscopy to the cecum or up to an obstructing carcinoma if present. Otherwise, an incomplete or technically unsatisfactory examination (such as poor bowel preparation) was repeated or excluded from analysis. A diagnosis of non-bleeding hemorrhoids was categorized as a negative result. All lesions were described, biopsied or removed. The location (right-sided, from the cecum to the splenic flexure; left-sided, the rest of the large bowel), size, number of lesions, and histological diagnosis of colorectal neoplasia were noted with reference to standard protocols. Size of neoplasia was estimated as the longest diameter of the lesion by the gap between two wings of fully opened biopsy forceps being 5 mm. Endoscopic resection or surgery was performed as indicated.

In this study, a pathologist with ten years of experience who was blinded to qlFIT results evaluated all the biopsied and resected tissues. The neoplasia of interest was classified into inflammatory polyp, hyperplastic polyp, adenoma and adenocarcinoma. Adenomas included tubular, tubulovillous, villous, or serrated types. Advanced adenomatous polyps (AAP) referred to those adenomas that were larger than 10 mm, or having at least 20% of villous histology, or any amount of high-grade dysplasia independent of size. All AAPs were re-examined to confirm the diagnosis. If the participants had more than one lesion of interest, the case was categorized based on the most histologically advanced lesion.

### Statistical analysis

Data were analyzed by SPSS statistical software, version 17.0 (SPSS Inc., Chicago, IL, USA). We employed t-test to compare quantitative data, and chi-square test to compare categorical variables. Colonoscopy findings and pathological diagnoses were the gold standard of this study. We calculated the diagnostic performance of three-sample qlFIT when one, two, or three positive tests were serving as the positivity threshold, respectively. Factors influencing positive qlFIT were analyzed through binary logistic regression. Patients' gender, age, location of the lesion, number of lesions and advanced histology (AAP and CRC) were brought into the analysis. A p value of less than 0.05 was considered of statistical significance.

## Results

### Participant Characteristics

513 patients participated this study and among them 293 (57.1%) were male. The mean age was 58.4±14.9 years. Complete colonoscopic insertion to the cecum was achieved in 491 patients (95.7%) in the first time, for the rest 22 patients, repeated colonoscopy examinations were all successful. Table 1 showed the baseline data of the study population.

**Table 1:** Demographic characteristics of the study population.

Variable	Number of participants (%) (N = 513)	
*Sex*		
Female	220 (42.9)	
*Age (years)*		
16–49	128 (25.0)	
50–59	126 (24.6)	
60–69	129 (25.1)	
70–79	108 (21.1)	
80 and older	22 (4.3)	
*Positive qlFIT(s)*		
None	253 (49.3)	
Once	103 (20.1)	
Two times	76 (14.8)	
Three times	81 (15.8)	
*Histology types of lesions*		
Cancer	52 (10.1)	
Advanced adenoma	70 (13.6)	
Non-advanced adenoma	51 (9.9)	
Hyperplastic polyp	15 (2.9)	
Inflammatory polyp	24 (4.7)	
	Any lesion	Advanced adenoma
	(N = 212)	(N = 70)
*Stratified by location*		
Left-sided	149 (70.3)	55 (78.6)
Right-sided	63 (29.7)	15 (21.4)
*Stratified by number*		
1	110 (51.9)	28 (40.0)
2	39 (18.4)	15 (21.4)
≥3	63 (29.7)	27 (38.6)
*Stratified by size (in diameter)*		
<1 cm	86 (40.6)	2 (2.9)
≥1 cm	126 (59.4)	68 (97.1)

### Diagnostic performance of three-sample qlFITs

**Figure 1:**
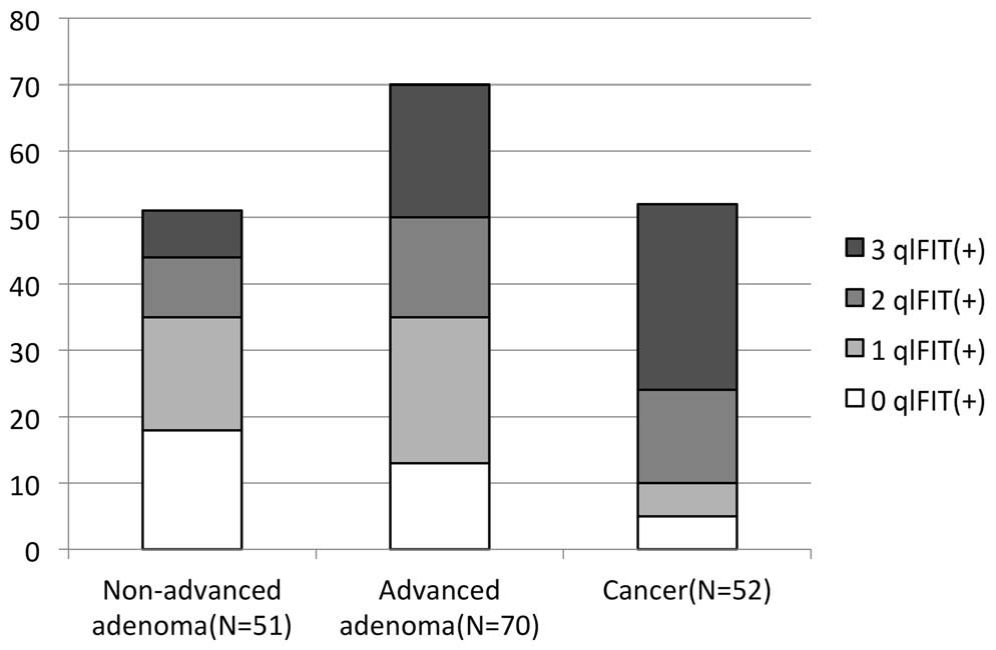
Distribution of positive qualitative fecal immunochemical tests in patients with colorectal adenoma or cancer.

In 290 (56.5%) patients colonoscopic examinations were normal. Another 11 (2.1%) participants had minor findings that were categorized into the normal group, including unspecific mild inflammation (n = 7), melanosis coli (n = 3) and subclinical benign stenosis (n = 1). In these 301 normal participants, 203 (67.4%) had no positive qlFIT, 51 (16.9%) had one positive qlFIT, 32 (10.6%) had two positive qlFITs, and 15 (5.0%) had three positive qlFITs. 18 patients with non-advanced adenoma, 13 with AAP, and 5 with CRC had no positive qlFIT. Figure 1 demonstrated the distribution of qlFIT results in patients with colorectal adenoma or cancer. Patients with polyps or cancer had significantly more positive qlFITs than normal participants (χ^2^ 114.55, P<0.001). Patients with CRC also had significantly more positive qlFITs than those with advanced adenomas (χ^2^ 13.26, P = 0.004). Between patients with advanced adenomas and those with non-advanced adenomas, the difference of positive qlFITs was not statistically significant (χ^2^ 6.38, P = 0.094). Table 2 and Table 3 summarized the diagnostic power of qlFIT for advanced adenomatous polyps (AAP) and CRC, respectively.

**Table 2:** Performance of qualitative fecal immunochemical test for advanced adenomatous polyps.

	1 qlFIT (+)	2 qlFITs (+)	3 qlFITs (+)
Sen % [95%CI] *	81.4 (57/70) [70.0–89.4]	50.0 (35/70) [37.9–62.1]	28.6 (20/70) [18.7–40.8]
Spe % [95%CI] *	54.2 (240/443) [49.4–58.9]	72.5 (321/443) [68.0–76.5]	86.2 (382/443) [82.6–89.2]
PPV % [95%CI] *	21.9 (57/260) [17.1–27.5]	22.3 (35/157) [16.2–29.8]	24.7 (20/81) [16.1–35.7]
NPV % [95%CI] *	94.9 (240/253) [91.2–97.1]	90.2 (321/356) [86.5–93.0]	88.4 (382/432) [84.9–91.2]
LR(+) [95%CI]	1.78 [1.53–2.07]	1.82 [1.37–2.40]	2.07 [1.34–3.21]
LR(−) [95%CI]	0.34 [0.21–0.56]	0.69 [0.55–0.87]	0.83 [0.71–0.96]

Sen: sensitivity; Spe: specificity; LR(+): positive likelihood ratio; LR(−): negative likelihood ratio; PPV: positive predictive value; NPV: negative predictive value.

*rates, absolute numbers, and 95% confidence intervals were provided.

**Table 3:** Performance of qualitative fecal immunochemical test for colorectal caner.

	1 qlFIT (+)	2 qlFITs (+)	3 qlFITs (+)
Sen % [95%CI] *	90.4 (47/52) [78.2–96.4]	80.8 (42/52) [67.0–89.9]	53.8 (28/52) [39.6–67.5]
Spe % [95%CI] *	53.8 (248/461) [49.1–58.4]	75.1 (346/461) [70.7–78.8]	88.5 (408/461) [85.1–91.2]
PPV % [95%CI] *	18.1 (47/260) [13.7–23.4]	26.8 (42/157) [20.2–34.5]	34.6 (28/81) [24.6–46.0]
NPV % [95%CI] *	98.0 (248/253) [95.2–99.2]	97.2 (346/356) [94.7–98.6]	94.4 (408/432) [91.7–96.3]
LR(+) [95%CI]	1.96 [1.71–2.23]	3.24 [2.63–3.98]	4.68 [3.28–6.69]
LR(−) [95%CI]	0.18 [0.08–0.41]	0.26 [0.14–0.44]	0.52 [0.39–0.70]

Sen: sensitivity; Spe: specificity; LR(+): positive likelihood ratio; LR(−): negative likelihood ratio;

PPV: positive predictive value; NPV: negative predictive value;

*rates, absolute numbers, and 95% confidence intervals were provided.

### Influencing factors of qlFIT

Among 212 participants diagnosed with colorectal polyps or cancer, 162 (76.4%) had at least one positive qlFIT. The results of qlFIT in subgroups categorized by potential influencing factors were presented in Table 4. Chi-square test revealed that positive qlFITs were significantly more often in patients with left-sided lesions and those with advanced histology (Table 4). Forward conditional logistic regression confirmed that left-sided location (OR 2.50, 95%CI 1.26–4.95) and advanced histology (OR 3.08, 95%CI 1.58–6.01) were independently associated to positive qlFIT results, but sex as female, age ≥60, and multiple lesions were not independent risk factors for positive qlFIT results.

**Table 4:** Comparison of qualitative fecal immunochemical test results in different categorization of patients with colorectal polyps or cancer.

Variables	Patients with at least one positive qlFIT (%) (N = 162)	Patients with negative qlFITs (%) (N = 50)	Odds ratios§ (95% CI)	*p*
*Sex*			0.81 (0.41–1.60)	0.519
male	99 (61.1)	28 (56.0)		
female	63 (38.9)	22 (44.0)		
*Age*			1.72 (0.85–3.50)	0.266
<60	48 (29.6)	19 (38.0)		
≥60	114 (70.4)	31 (62.0)		
*Location of lesions*			2.50 (1.26–4.95)	0.004
Left-sided	122 (75.3)	27 (54.0)		
Right-sided	40 (24.7)	23 (46.0)		
*Number of lesions*			1.54 (0.78–3.33)	0.102
1	79 (48.8)	31 (62.0)		
≥2	83 (51.2)	19 (38.0)		
*Histology of lesions*			3.08 (1.58–6.01)	<0.001
Advanced histology*	104 (64.2)	18 (36.0)		
Non-advanced histology	58 (35.8)	32 (64.0)		

*Advanced histology referred to advanced adenomatous polyps and colorectal cancer.

§ Forward conditional logistic regression controlling for sex as female, age ≥60, left-sided location, multiple lesions and advanced histology. 95% CI: 95% confidence interval.

## Discussion

Qualitative fecal immunochemical test (qlFIT) is one of the most commonly used noninvasive tests in clinical practice. In this study we extensively evaluated the performance of repeated qlFIT in screening for colorectal neoplasia among high-risk outpatients in a tertiary medical center. Despite the steady increase of CRC in China over the last decades, screening for CRC has proved difficult in this country because of poor compliance with colonoscopy examination, particularly in those of lower socioeconomic status [2], [3], [7]. A similar inequity in the access to CRC screening was also present in the United States, probably due to disparity of income and restricted colonoscopy service [19]. Therefore, to improve screening participation while maximizing the utility of healthcare resources, we need a simple, noninvasive and reasonably accurate method, such as fecal occult blood test (FOBT), to identify those high-risk people who are most likely to benefit from colonoscopy examination. Many investigators have acclaimed that using FOBT in CRC screening can reduce the incidence and mortality of CRC, as well as saving medical resources by postponing unnecessary colonoscopy examinations [8]–[12], [20], [21]. But most of such studies focused on average-risk population. The major interest of our study, however, is centered on opportunistic screening in high-risk patients with inadequate colonoscopy services.

With abundant evidence supporting the use of FOBT in CRC screening, the question is no longer whether FOBT qualifies a test option, but which FOBT to employ and how to optimize its use. Traditional guaiac-based test is faulted for its inadequate power to detect advanced adenomas [17], [18], [20], [21]. A majority of recent studies focused on quantitative tests (qnFITs) in CRC screening [9], [11], [17], [22], [23]. Since qnFIT is more costly while less available than qualitative FIT (qlFIT), comparing diagnostic performance between these two methods is of clinical and economic significance. In our study, the sensitivity of three-sample qlFIT to detect advanced adenomatous polyps was 28.6% to 81.4% and specificity was 54.2% to 86.2%, depending on the positivity thresholds. The likelihood ratio of three positive qlFITs was 2.07 for advanced adenomas and 4.68 for CRC, respectively. Our results are comparable to those of other investigations. For instance, in the study by Haug et al, the sensitivity of quantitative FOBTs for advanced adenomas was 33% at a specificity of approximately 95%. They also proved that the sensitivity of quantitative FOBTs was very close to those of qualitative tests at strongly divergent levels of specificity [8]. Levi et al measured the hemoglobin content of three bowel movements, compared the highest value to colonoscopy findings, and revealed that this method had the sensitivity of 67% and specificity of 91.4% for colorectal neoplasia [13]. Chiang et al found that single qlFIT had the sensitivity of 24.3% and specificity of 89.0% to detect neoplasia in the lower gastrointestinal tract [15]. Ou et al pointed out that both qnFIT and qlFIT are superior to guaiac test, and qnFIT was slightly better than qlFIT in terms of higher positive LR (3.7 versus 3.3) to detect advanced colorectal adenoma [17]. Our study results demonstrated that, if used appropriately, repeated qlFIT has a similar performance to qnFIT in screening for clinically significant colorectal neoplasia.

Some authors used multi-sample FOBT to detect colorectal lesions, based on the assumption that small adenomatous polyps do not tend to bleed and cancers or large polyps may bleed intermittently, thus repeated measurements should be more sensitive than a single test [11]–[14]. But others argued against this approach by showing that increasing samples did not carry about extra screening efficacy [24]. Our study has three findings in favor of the strategy of multi-sample tests. First, we showed that when three qlFITs were obtained, about a half of patients with colorectal adenoma and a third with cancer had at least one negative result, suggesting that single test may be falsely negative and overlook these lesions consequently. Second, when three positive qlFITs served as positivity threshold instead of single one, the positive likelihood ratio for CRC increased from 1.96 to 4.68, and positive predictive value elevated from 18.1% to 34.6%, while the negative predictive value slightly decreased from 98.0% to 94.4%, implying that consecutive positive qlFIT is a better predictor for CRC than single test. Last but not least, in 253 patients with all three tests being negative, only 5 patients were with CRC and 13 with AAP. Colonoscopic examination is unnecessary and could have been postponed in 92.9% (235/253) of participants of this group. Therefore, even in high-risk population such as those having symptoms of lower gastrointestinal tract, a family member diagnosed with CRC, or a history of adenomatous polyps, repeated negative qlFIT is nevertheless strongly against the possibility of significant colorectal neoplasia. A major advantage of three-sample test, as we have shown above, is to offer higher specificity and positive predictive value than single test, which is important to reduce false positive results. Some drawbacks, however, should also be considered before using it in mass screening for CRC. For instance, multi-sample test incurs more labor and cost than single assay, and its sensitivity and specificity could change in the setting of population-wide surveillance. Therefore we believe that further investigations are necessary to assess the screening power of multi-sample qlFIT, particularly in the general population with average risk of CRC. After all, three-sample qlFIT test provides clinicians with a range of likelihood to determine the necessity of colonoscopy examination. If all three tests are positive, a following colonoscopy is indicated. Conversely, when all three tests are negative and no other diagnostic clue is present, it is relatively safe to postpone colonoscopy examination. When one or two tests are positive, clinicians should make individualized decisions in light of other information such as patient risk profile and available colonoscopy resources. A major advantage of qnFITs is its flexibility to adjust the positivity threshold in accordance to variable circumstances, but such flexibility, as we have showed above, can also be obtained through the application of multi-sample qlFIT.

Furthermore, qlFIT has much lower expense than qnFIT. Although multi-sample qlFIT costs more than single test, it is remarkably cheaper than qnFIT nevertheless. In the study by Wilschut JA the cost of one-sample qnFIT was €19.22 (€14.85 for test kit and €4.37 for personnel and material), which is about $26 [10]. In another study performed by Goede SL, one-sample qnFIT incurred a similar expense of €19.88 (about $27) [24]. But in our study each measurement of qlFIT only cost $2.5. Deng et al showed that cost, embarrassment, and fear of complications were major obstacles preventing people from participating screening for CRC, especially in developing areas [7]. In light of the rapid increase of CRC in populations with low to moderate income worldwide, an affordable screening protocol should be of help to improve the compliance with CRC screening and bring cost under control. For instance, according to the statistics of Chinese central government, the annual income of a Chinese farmer in 2012 was $1309 (about $ 3.6 per day) on average. To screen for CRC in such a vast and medically underserved population, the fecal occult blood test needs to be simple, cheap and reasonably accurate. Based on the data of this study, we believe that more studies are needed to explore the potential of qlFIT to fulfill these requirements.

It has been controversial whether anatomical sites of colorectal neoplasia influence the sensitivity of FOBTs. de Wijkerslooth et al found a similar sensitivity of fecal immunochemical test for proximal and distal advanced neoplasia of the large intestine [25]. But in our study, the left-sided location was independently associated with positive qlFIT results. A systematic review supported our findings by showing that FOBT was more sensitive for left-sided versus right-sided colorectal neoplasia [26]. The study by de Wijkerslooth et al was an invitational primary screening program and the prevalence of advanced neoplasia (adenomatous polyps and colorectal cancer) was only 9%, much lower than that of our study. The remarkable difference of study population may account for the discrepancy between our findings. A possible explanatory model for the higher sensitivity of FOBT for lesions in the distal colon is as follows: a certain proportion of advanced neoplasms present a relatively strong source of bleeding regardless of anatomical sites. But another proportion of advanced adenomas is a weak source of bleeding. They do not yield positive FOBTs unless the trace fecal hemoglobin amount up to a clinically relevant cutoff level before degrading. Since the time of stool passing distal colon is much shorter than from the proximal part, the likelihood of left-sided neoplasia to be detected through FOBT is thus increased. The study by Haug et al supports our speculation by showing that weak sources of bleeding were more frequently detected in the left colon than in the right colon [27]. The lower sensitivity of FOBT for right-sided neoplasia has clinical relevance. Baxter has questioned colonoscopy regarding protection from right-sided neoplasia [28]. Our study supports the hypothesis that FOBT also has lower performance for right-sided tumors. This will raise the issue of site-specific performance of the screening program and the necessity to re-evaluate the current strategy that combines flexible sigmoidoscopy and FOBT.

In addition, we have confirmed the correlation between advanced neoplasia pathology and positive qlFITs, suggesting that consecutive positive qlFITs are predictive for severe colorectal lesions, such as advanced adenoma and cancer, thus prompt a following colonoscopy examination. This finding is consistent with that of Rozen P and Digby J in that fecal occult blood is related with the severity of colorectal neoplasia [14], [23]. A possible explanation is that when compared to polyps without villous histology or dysplasia, advanced colorectal adenomas tend to be of larger size and richer blood supply, thus are more readily to bleed.

The strengths of this study are its resemblance to the “real world” practice and that all participants received colonoscopy, which allowed for detailed evaluation of multi-sample qlFIT in a population with relatively high risk of CRC. Our study has some limitations. Perhaps the most noticeable one is the highly selected study population. Because this is a retrospective study performed in a tertiary medical center, to avoid referral bias is difficult. In addition, our study participants were a heterogeneous mixture of CRC risk, so one should be cautious to generalize our conclusion to average-risk populations. With these shortcomings in mind, we believe nonetheless that our study provides some useful findings that merit further investigation. In screening for CRC among average-risk people, we expect that the effect of multi-sample qlFIT would be comparable to that of quantitative tests, but at lower cost.

In conclusion, three-sample qlFIT of separate bowel movements has reasonably good performance to detect CRC and, more importantly, advanced adenomatous polyps in gastrointestinal outpatients. It's necessary to do further research in other populations to validate this screening strategy.
